# Effect of Gutta-Percha Removal Methods on Fiber-Post Bond Strength

**DOI:** 10.3390/dj14010038

**Published:** 2026-01-06

**Authors:** Abdul Rahman Hamwieh, Haitham Elbishari, May Aljanahi, Fatemeh Amir-Rad, Amre R. Atmeh, Moosa Abuzayeda, Amar H. Khamis, Rashid El Abed

**Affiliations:** 1 Hamdan Bin Mohammed College of Dental Medicine, Mohammed Bin Rashid University of Medicine and Health Sciences, Dubai P.O. Box 505055, United Arab Emiratesamar.hassan@dubaihealth.ae (A.H.K.); rashid.elabed@dubaihealth.ae (R.E.A.); 2 School of Medicine, Dentistry and Biomedical Sciences, Queen’s University Belfast, Belfast BT9 7BL, UK; 3 The Faculty of Biology, Medicine and Health, The University of Manchester, Manchester M23 9LT, UK

**Keywords:** fiber posts, root dentin, gutta-percha removal techniques, push-out bond strength, adhesive failure

## Abstract

**Objectives:** This study evaluated the effect of three root canal filling material (RCFM) removal techniques—mechanical, thermo-mechanical, and chemico-mechanical—on the micro push-out bond strength of fiber posts to root dentin in endodontically treated teeth. **Materials and Methods:** Forty-five single-rooted human premolars were endodontically treated and randomly allocated into three groups (*n* = 15) according to the RCFM removal technique used during post-space preparation: mechanical, thermo-mechanical, or chemico-mechanical. Fiber posts were luted using a dual-cure resin cement. Roots were embedded in resin and sectioned into coronal, middle, and apical thirds. Micro push-out bond strength was measured using a universal testing machine. Failure modes were examined under a stereomicroscope and validated using scanning electron microscopy. Statistical analysis used two-way ANOVA and Chi-square tests (α = 0.05). **Results:** Both the thermo-mechanical and mechanical groups showed significantly higher bond strength values than the chemico-mechanical group (*p* < 0.001). Across all groups, the coronal third recorded the highest bond strength, while the apical third presented the lowest values (*p* < 0.001). Adhesive failure at the dentin–cement interface was the most frequent failure mode. **Conclusions:** The gutta-percha removal technique and the root canal region significantly influence fiber-post bond strength. Solvent-based chemico-mechanical methods may adversely affect adhesion quality. **Clinical Relevance:** Thermo-mechanical and mechanical removal techniques may provide more reliable post retention during retreatment procedures, improving adhesion and reducing the risk of post debonding in daily practice.

## 1. Introduction

Resin composites are increasingly used to restore endodontically treated teeth due to their bonding ability and improved physical properties. In cases with significant tooth loss, fiber posts are required to retain the core, offering dentin-like elasticity, optimal aesthetics, durable bonding with composites, biocompatibility, corrosion resistance, and reduced treatment time [1]. Survival rates for teeth restored with post and core systems range from 85% to 95% over 5–10 years, with fiber posts performing favorably when combined with adhesive resin cements [2,3]. However, failures such as loss of retention and post fracture remain the most reported complications [4,5].

The long-term success of fiber posts is highly dependent on the integrity of the adhesive interfaces between dentin, resin cement, and the post, as any disruption in bonding can significantly compromise retention. Research has shown that the composition of the post–resin matrix plays a key role in determining the strength of the post–cement interface, directly influencing post stability and survival [6]. Similarly, investigations into silane coupling agents have demonstrated that appropriate surface treatments can improve the adhesion between fiber posts and resin composites, highlighting the importance of optimizing adhesive interfaces for durable outcomes [7]. A major clinical challenge, however, arises during endodontic retreatment and post space preparation, where removal of existing root canal filling materials (RCFM) is required. Residual gutta-percha or sealer can act as a barrier to bonding, resulting in microleakage and restoration failure [8]. Several methods have been used to remove RCFMs, yet each presents notable drawbacks. Mechanical approaches with rotary instruments risk excessive dentin removal and potential perforation, particularly in narrow or curved canals [9]. Thermal techniques effectively soften gutta-percha but may produce heat levels that jeopardize dentin integrity and periradicular tissues [10]. Chemical solvents such as chloroform and eucalyptus oil enhance removal efficiency, but residual traces may compromise bonding and pose cytotoxicity concerns [11]. Achieving thorough filling removal while preserving dentin thickness is therefore essential to maintain root strength and minimize fracture risk [12]. These challenges highlight the need for optimized retreatment protocols that balance efficiency with biological safety to ensure reliable adhesive outcomes. Accordingly, this in vitro study evaluated the micro push-out bond strength of fiber posts cemented with Bis-GMA-based resin after different gutta-percha removal techniques: mechanical, thermal-mechanical, and chemical-mechanical. Despite the widespread use of various gutta-percha removal techniques, the literature lacks clear comparative evidence on their differential effects on fiber-post bond strength and adhesive interface integrity, leaving an important gap regarding how the choice of removal method may influence long-term clinical outcomes. The null hypothesis stated that neither the removal technique nor the root canal region would significantly affect bond strength or failure mode.

## 2. Materials and Methods

This study was conducted in accordance with the principles of the Declaration of Helsinki and was approved by the Research Ethics Committee at Hamdan Bin Mohammed College of Dental Medicine, Mohammed Bin Rashid University of Medicine and Health Sciences, Dubai Health (IRB #: MBRU IRB-2023-98). Written informed consent was obtained from all patients prior to tooth extraction, and additional consent was obtained for the use of extracted teeth in research.

### 2.1. Sample Size Calculation

Based on a previous study by (Zare Jahromi et al., 2017) [13], the sample size Calculation of sample size for ANOVA is

$$n=\frac{N\delta^{2}}{\sum_{i=1}^{k}{\left(\mu_{i}-\mu \right)}^{2}(1-\rho )}$$

where *n* is the required sample size. N is the total number of observations or subjects in the entire study (across all groups).

δ^2^ is the population variance.
μ_i_ is the mean of the i-th group.
μ is the overall mean of all groups.
k is the number of groups or treatments.
ρ is the intra-group correlation (if your data has repeated measures or is not independent, this accounts for the correlation between observations within the same group).

The sample size = 42~45 (15 per group).

### 2.2. Sample Selection

**Figure 1:**
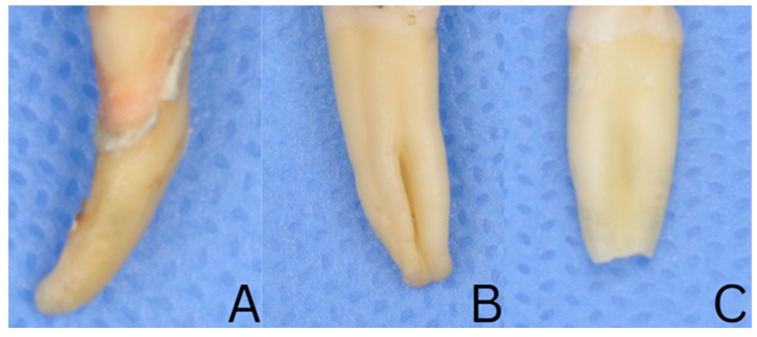
Excluded samples. (**A**): Curved root more than 15 degrees, (**B**): multirooted tooth, (**C**): open apex.

All samples were screened using periapical radiographs in facial and proximal projections to confirm the presence of a single and straight or mildly curved canal, along with a fully formed apex, and normal anatomy. Teeth with a root curvature exceeding 15° (Schneider method) or accessory canals were excluded. Crowns were removed, and root lengths standardized to 16 ± 1 mm. The mesiodistal and buccolingual dimensions at the cementoenamel junction (CEJ) were measured with a digital microcaliper, and only teeth within a ±10% tolerance of the mean values were included to ensure uniform morphology (Figure 1). Additionally, each root was examined under a Leica M320 (Leica Microsystems, Wetzlar, Germany) dental microscope (×10 magnification) to confirm the absence of cracks, fractures, or structural defects.

### 2.3. Preparation and Root Canal Treatment of Samples

The cleaned specimens were disinfected by immersion in 2.5% sodium hypochlorite for 2 h. Following disinfection, the teeth were stored in 0.1% sodium azide (NaN_3_) solution at room temperature until further use.

**Figure 2:**
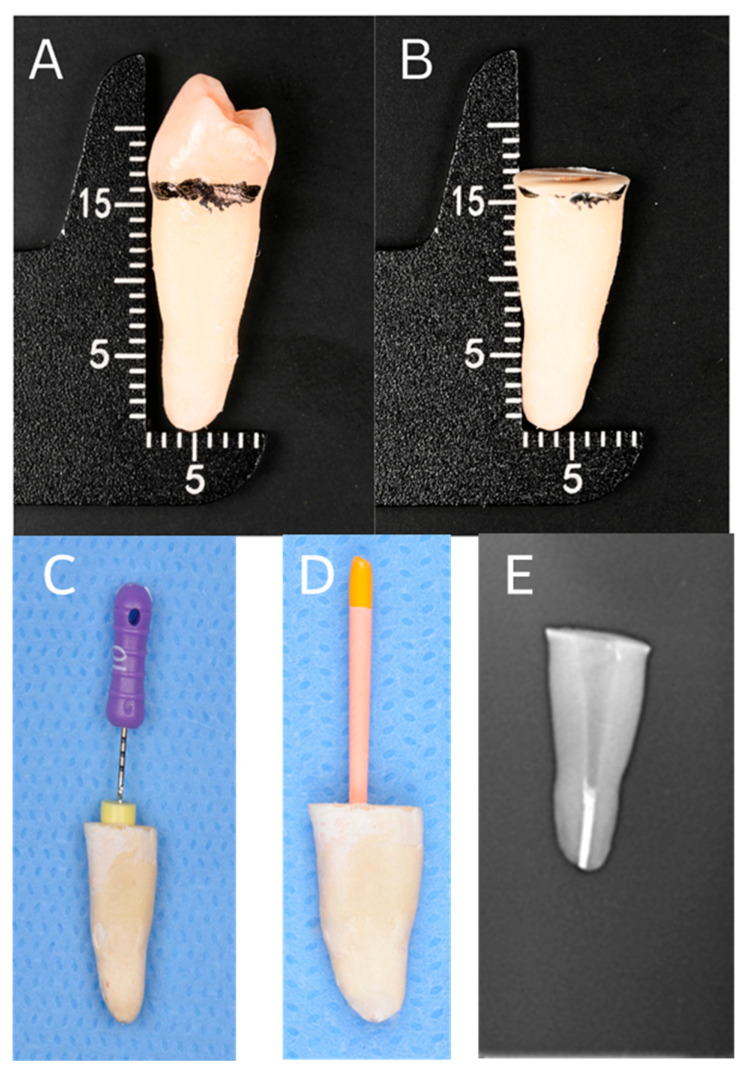
Sample preparation steps. (**A**): Before the de-coronation, (**B**): root length after crown de-coronation (16 ± 1 mm), (**C**): establishment of working length, (**D**): master cone fitting/obturation, (**E**): radiograph of obturation.

The crowns of all teeth were decoronated then sectioned 2 mm coronal to the cementoenamel junction (CEJ) on a plane perpendicular to the long axis of the tooth using a fissure carbide bur (Komet-Brasseler GmbH, Lemgo, Germany) mounted on a high-speed handpiece with air–water spray. Root lengths were standardized to 16 ± 1 mm, and the cut surfaces were smoothened using a fine diamond disc (Komet-Brasseler GmbH) (Figure 2A,B).

Canal patency was confirmed with a size 15 stainless steel K-file (Dentsply Maillefer, Ballaigues, Switzerland) inserted to the apical foramen (AF) while using an operative dental microscope 10× magnification. The working length (WL) was established as 0.5 mm short of the AF (Figure 2C).

Instrumentation was performed by a single operator using size 50 R-Motion files of 4% taper (FKG Dentaire, La Chaux-de-Fonds, Switzerland) in a reciprocating motion mode of an X-Smart Plus motor (Dentsply Sirona, Ballaigues, Switzerland). The files were used with gentle 2–3 mm strokes, applying light apical pressure until the working length was reached. During instrumentation, irrigation with 5.25% sodium hypochlorite (NaOCl) was performed by #30-gauge IrriFlex (PD Produits Dentaires SA, Vevey, Switzerland) needle. After preparation, canals were rinsed with distilled water and dried with size 50 paper points (Dentsply Maillefer, Ballaigues, Switzerland).

The root canals were obturated using the warm vertical compaction technique with gutta-percha and a resin-based sealer (AH Plus; Dentsply DeTrey GmbH, Konstanz, Germany). A master cone was fitted to working length with confirmed tug-back, and a thin layer of sealer was applied to the canal walls before placement (Figure 2D). A controlled down-pack was performed to create a dense apical plug, followed by thermoplasticized gutta-percha backfill to obturate the remaining canal space. Post-obturation periapical radiographs were taken to confirm the quality and density of the filling (Figure 2E). Excess gutta-percha was removed, and the coronal mass was compacted with a plugger. The access cavities were sealed with a small cotton pellet and Cavit (3M ESPE, St. Paul, MN, USA).

### 2.4. Post Space Preparation and Luting Fiber Post

All samples were stored in an incubator (DAIHAN Incubator, Gravity Convection-type, “WIG”, Gangwon, Republic of Korea) at 100% humidity and 37 °C for 30 days to allow the sealer to fully set before initiating the removal of RCFM. After the incubation period, the specimens were coded and randomly assigned to three groups (*n* = 15) based on the removal method of RCFM using a Web-based algorithm (www.random.org) accessed on 13 October 2024 before placing fiber posts. The first group was mechanical (M), the second group was thermo-mechanical (TM), and the third group was chemico-mechanical (CM). In the M group, the RCFM was removed using different sizes of drills (Paseo reamers). The first step included the use of a universal drill (RelyX Fiber Post, 3M ESPE, St. Paul, MN, USA) to remove the RCFM to the working length of 10 mm, leaving 5 mm of RCFM apically to mimic clinical scenarios. In the TM group, the RCFM was removed using a heat source fitted with a medium-fine endodontic heated tip (Elements Free Obturation System-Kerr, Orange, CA, USA) activated at 200 °C. The plugger was applied buccally and lingually to the RCFM at 10 mm for 5 to 8 s, allowing for thermal softening and displacement of the material. In the third group (CM), RCFM was dissolved using eucalyptus oil (Eucalyptol Cerkamed, Stalowa Wola, Poland), and removed using different sizes of H-files. After RCFM removal using different techniques, the canals were irrigated with 0.9% Sodium Chloride solution and further enlarged to prepare a post space using drill size 1 (RelyX Fiber Post, 3M ESPE, St. Paul, MN, USA). All samples were radiographically examined to confirm the absence of residual RCFM prior to post cementation.

After the final irrigant application, paper points were used to dry the prepared canals. The dual-cure resin cement (RelyX Unicem, 3M ESPE, St. Paul, MN, USA) was delivered into the post space for each prepared sample as per the manufacturer’s guidelines. Fiber post size 1 (1# Fiber Post, 3M ESPE) was seated and held in place using finger pressure. Excess resin cement was removed with a microbrush. The posts were light-cured for 40 s from the buccal and lingual sides, then for 40 s each with the curing unit tip angled 45 degrees to the post’s long axis (at 600 mW/cm^2^). A composite resin was used to seal the coronal part and was light-cured. Samples were preserved in saline at 100% humidity and 37 °C for 7 days to ensure complete cement setting.

### 2.5. Fabrication of Experimental Sample

**Figure 3:**
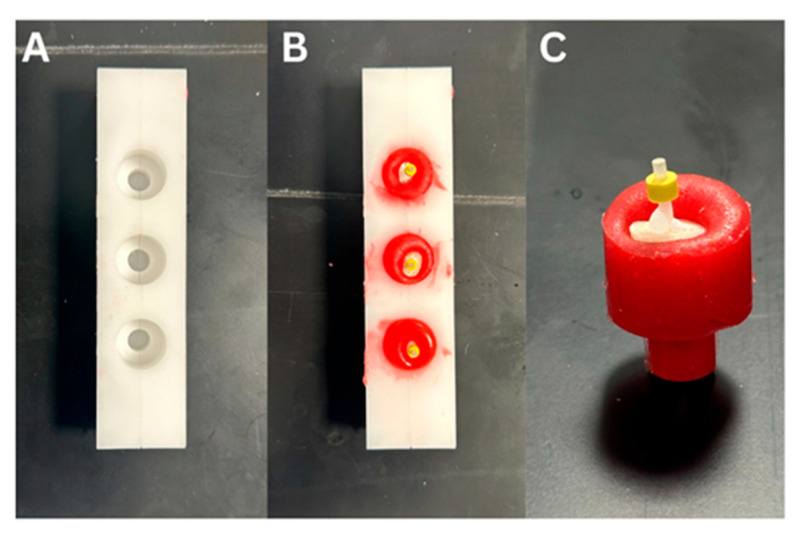
(**A**): Custom-fabricated plastic mold, (**B**): samples embedded in the pattern resin, (**C**): sample held by the resin jig.

A custom-made plastic mold was designed in order to hold the specimens. Petroleum gel was placed in the inner wall of the mold as a separating medium in which the mold was filled with pattern resin (GC America-Pattern Resin LS 1-1 PKG, Alsip, IL, USA). The sample was placed perpendicular and at the center of the pattern resin until the apical 1–2 mm of the core material was covered by the acrylic resin (Figure 3). Each specimen was placed in the saw machine (Buehler, IsoMetTM 1000, 713-IPS-04427, Lake Bluff, IL, USA) perpendicular to the long axis of the saw disc and the sample was cut into 2 mm sections with a cutting blade thickness of 0.3 mm. The blade was calibrated to zero at the coronal end of the core to allow cutting at a distance of 0.7 mm with a speed of 275 rpms and a load of 100 g, following previously described methods [7].

**Figure 4:**
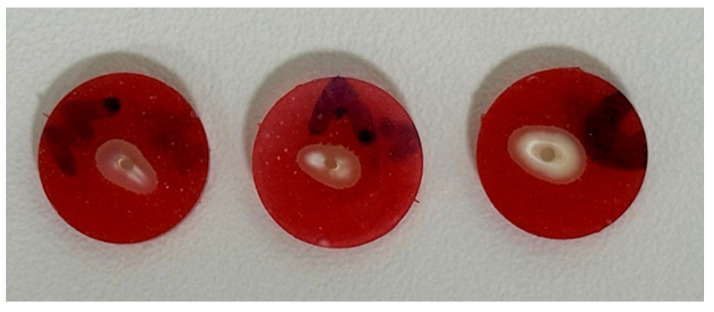
Three labeled sections of a single root, representing the apical, middle, and coronal thirds for reference.

A 0.7 mm segment was first sectioned from the coronal end of the composite core and excluded from analysis. Thereafter, three consecutive slices, each measuring 2.0 mm in thickness, were prepared. These three specimens were designated for bond strength testing (Figure 4).

The thickness of each slice was measured using a digital caliper (Insize digital caliper, Standard Model, 1108-150, 0-6”/150 mm, Boituva-SP, Brazil) to confirm accuracy, and the value was recorded. The coronal surface of each section was marked with waterproof marker to identify the coronal and apical surfaces of each section and any sharp edges in the specimen were removed with a low-speed polishing disk.

### 2.6. Assessment of the Push-Out Bond Strength

**Figure 5:**
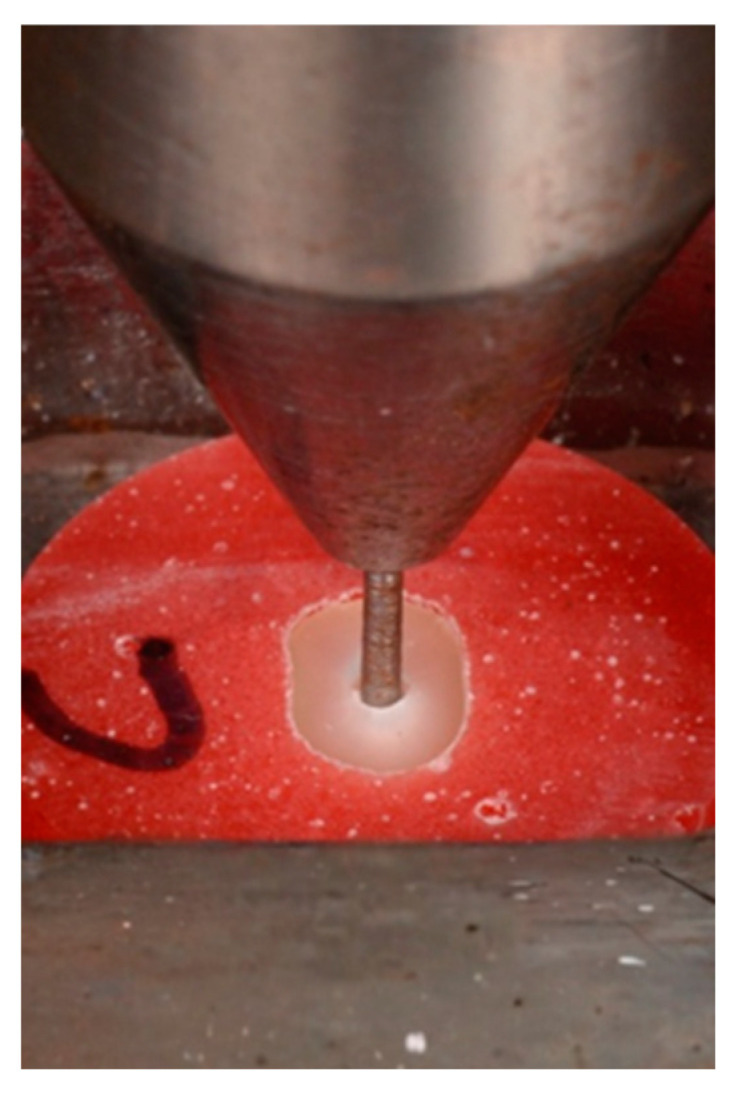
A specimen undergoing the push-out test with a custom pin aligned centrally to the fiber post in an apical–coronal direction.

**Figure 6:**
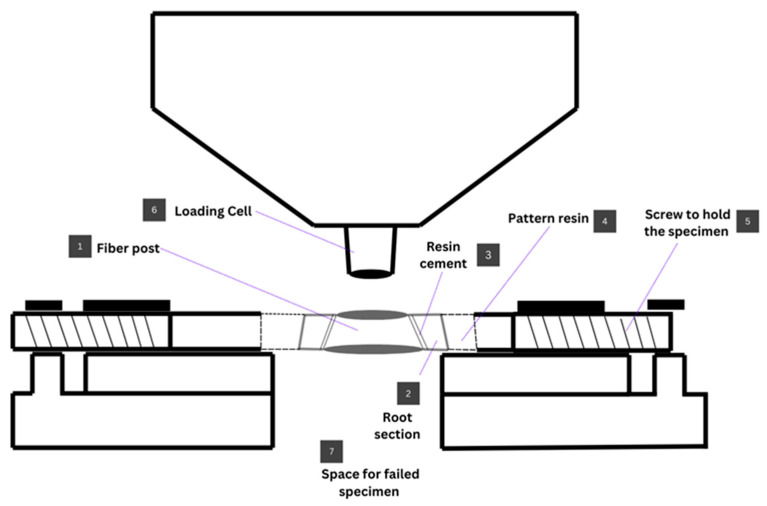
Schematic drawing of the push-out testing set up. (1) Fiber post; (2) root section; (3) resin cement; (4) pattern resin jig holding the specimen; (5) screws to hold and center the sample to the pin; (6) loading cell centered to the post.

Push-out bond strength was measured using a universal testing machine (Universal Testing Machine M350-5CT, Testometric, Roshdale, UK). Each section was placed on a custom-made stainless steel base. A push-out pin of 0.7 mm diameter was attached to the loading cell of the testing machine. The push-out pin was positioned over the center of the post, so that the force was applied to the post surface without stressing the surrounding post space. A constant load was applied in the apical–coronal direction of each section at a crosshead speed of 0.5 mm/min (Figure 5 and Figure 6).

The peak force at the time of post segment extrusion from the section was taken as the point of bond failure, and the value was recorded in Newtons (N). Bond strength in megapascals (MPa) was then measured by the calculated surface area using the formula of Bond strength (MPa) = Load value, recorded in N/Area of the bonded interface. Bonded surface area = 2πrh, where π is constant, r is the radius of the post, and h is the height of the sample in mm. This testing method was adapted from previously described studies [6].

### 2.7. Mode of Failure

All samples were viewed under a stereomicroscope (OPTIKA Stereomicroscopes, Ponteranica, Italy) with a magnification of ×35. Modes of failure were divided into 4 types as described in Table 1. Two examiners determined the mode of failure. The first examiner assessed all samples twice, with a 2-week interval, and the coefficient of variation was calculated based on these repeated measurements. Inter-examiner variability was also evaluated by comparing measurements between the two examiners. Both examiners underwent standardized training and calibration before the assessments to ensure consistency in failure mode evaluation. Two samples from each group were sputter-coated with a thin layer of gold, mounted on stubs, and examined using scanning electron microscopy (SEM) with a magnification of more than ×500 to study failure modes and the surface morphological structure.

### 2.8. Statistical Analysis

Data was collected and analyzed using the Statistical Package for Social Sciences (SPSS, version 29, IBM Corp., Armonk, NY, USA). The push-out bond strength data in MPa were analyzed using two-way analysis of variance (ANOVA). If a significant difference was found between groups, the differences were revealed using Post Hoc test -Tukey HSD. A *p*-value of ≤0.05 was considered statistically significant. Failure mode analysis was analyzed using Chi-square test. The intra-examiner and inter-examiner agreement for mode of failure was assessed using Intra-class Correlation Statistics.

## 3. Results

**Figure 7:**
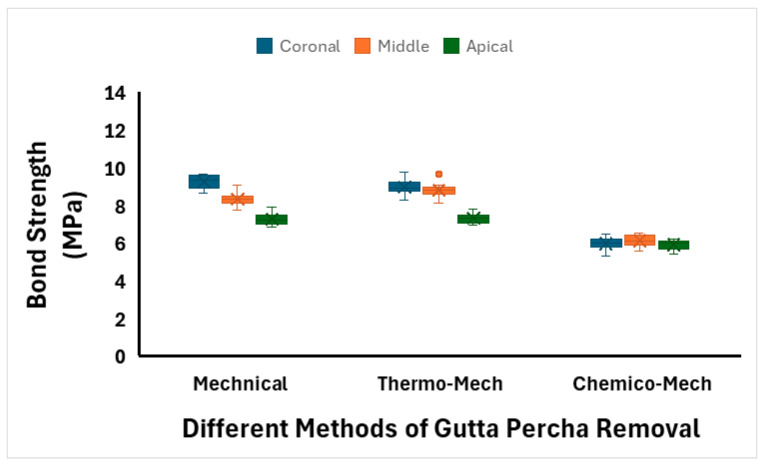
Box plots of push-out bond strength (in MPa) of different methods of RCFM removal at three different root sections.

The means and standard deviations of push-out bond strength (MPa) of all tested groups are summarized in Table 2 and Figure 7. Among all the groups, teeth in the TM group exhibited the highest bond strength (8.37 ± 0.81 MPa) for the cemented posts, followed by the M method (8.29 ± 0.88 MPa). The CM method resulted in the lowest bond strength (6.01 ± 0.30 MPa). Regarding root sections, the bond strength was highest in the coronal section (8.06 ± 1.54 MPa), followed by the middle section (7.77 ± 1.21 MPa), and was lowest in the apical section (6.84 ± 0.71 MPa).

A two-way ANOVA was performed to assess the effects of RCFM removal methods and root sections on push-out bond strength and the effect of their interaction. The analysis revealed a significant main effect of the RCFM removal method on bond strength (*p* < 0.001), a significant main effect of root section (*p* < 0.001), and a significant interaction effect between method and section (*p* < 0.001).

Post hoc Tukey tests showed no significant difference between the M and TM methods (*p* = 0.482). However, both M and TM methods showed significantly higher bond strength than the CM method (*p* < 0.001) (Table 3). For root sections, all pairwise comparisons between coronal, middle, and apical sections were statistically significant (*p* < 0.001) (Table 3).

**Figure 8:**
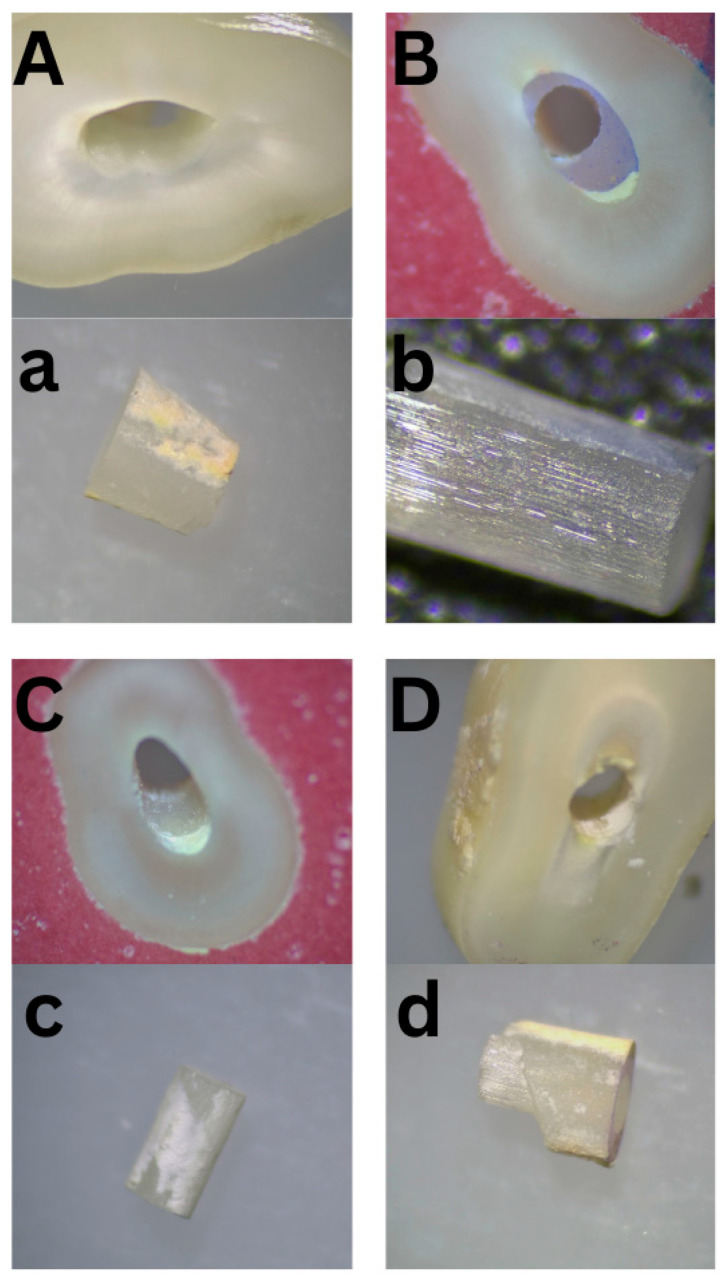
Types of failure modes captured using stereomicroscope showing (**A**): Adhesive T-C: Minimal cement remnants are observed on the inner root surface, while the cement almost entirely covers the fiber-post surface (**a**). (**B**): Adhesive C-P: The cement fully covers the inner root surface with little to no remnants on the fiber post (**b**). (**C**): Cohesive: Cement is present on both the inner root surface and the fiber post (**c**). (**D**): Mixed: A combination of adhesive T-C and C-P failures, where the cement covers approximately 50–60% of both the inner root surface and the post (**d**).

A total of 135 valid root sections were evaluated for mode of failure. The distribution of failure modes is presented in Table 4. Adhesive T-C failure was the most common, accounting for 44.4% of cases; on the other hand, adhesive P-C and mixed failure were the least common, accounting for 14.8% for each of them. Figure 8 illustrates various failure modes, capturing all types of failures on the dentin and failed post surfaces using a stereomicroscope (OPTIKA Stereomicroscopes, Ponteranica, Italy).

**Figure 9:**
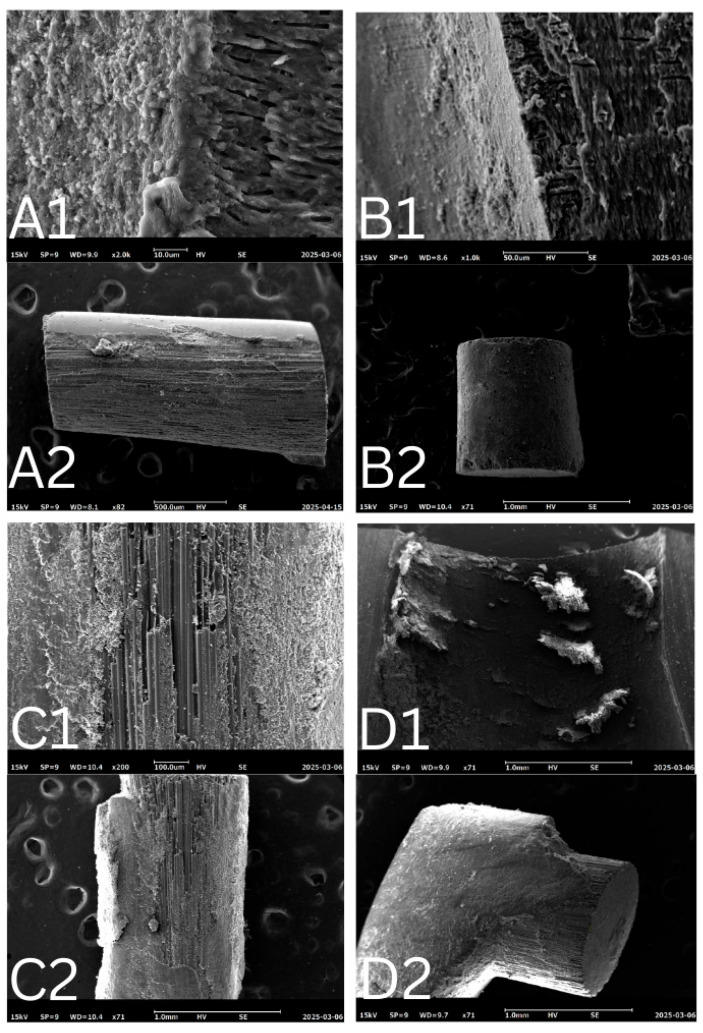
Scanning electron microscopy (SEM) images were captured at magnifications > 500. In image set A, the inner root surface (**A1**) is almost entirely covered with cement, while the corresponding post surface (**A2**) shows minimal cement remnants, indicating (C–P) adhesive failure. Image set B displays the opposite pattern: the root surface (**B1**) shows little cement, while the post (**B2**) is fully coated, representing (T–C) adhesive failure. In set C, both the root surface (**C1**) and the post (**C2**) are covered with cement, consistent with cohesive failure within the cement layer. Finally, image set D shows partial cement coverage of approximately 50–60% on both the root surface (**D1**) and the post (**D2**), characteristic of a mixed failure mode.

Intra-examiner agreement was assessed by comparing the first and second readings by the same examiner, two weeks apart. The Cohen’s Kappa value was 0.804 (*p* < 0.001), indicating substantial to almost perfect agreement. Inter-examiner agreement between Examiner 1 and Examiner 2 showed a Cohen’s Kappa value of 0.332 (*p* < 0.001), reflecting fair agreement. The SEM images strongly supported the visual assessments using stereomicroscope, offering clear confirmation and consistency in identifying the failure modes of the samples (Figure 9).

The association between the mode of failure and the method of RCFM removal was analyzed. There was no significant association observed (Kappa = −0.034, *p* = 0.492) (Table 4). Additionally, the distribution of failure modes across root sections revealed a weak but statistically significant association between root section and mode of failure (Kappa = 0.110, *p* = 0.028) (Table 4).

## 4. Discussion

This in vitro study aimed to evaluate the micro push-out bond strength of fiber posts luted with a dual-cured resin cement to single-rooted teeth following the use of three different gutta-percha removal techniques: mechanical, thermo-mechanical, and chemo-mechanical. The bond strength was assessed at three distinct root levels: coronal, middle, and apical. The results demonstrated no significant difference in bond strength between the mechanical and thermo-mechanical techniques. However, both showed significantly higher bond strength compared to the chemo-mechanical method. Additionally, a statistically significant difference in bond strength was observed among the different root regions, with the highest values recorded in the coronal third. Based on these findings, the first null hypothesis was partially rejected, and the second was fully rejected, confirming that both the removal method and root level significantly influence fiber-post retention.

Several methods have been used for bond strength analysis, including tensile, micro-tensile, pull-out and push-out tests [14]. Furthermore, the push-out test is considered less stressful on the bonding interface than other tests, making it particularly suitable for evaluating the retention of fiber posts [15,16]. The push-out test was selected in the present study due to its technical advantages, namely, its ability to provide a more uniform stress distribution across the adhesive interface, minimize specimen loss, and facilitate ease of execution [17].

In this study, all specimens consisted of caries-free, single-rooted premolars with standardized root lengths (≥16 mm). The teeth were decoronated at the CEJ and subjected to root canal treatment. During post space preparation, the drills inevitably generated a smear layer composed of gutta-percha remnants, sealers, and inorganic particles, which adversely affect the bond strength between the Fober post and root dentine by impeding adhesive penetration into the dentinal tubules [18,19].

In the present study, the mechanical and thermo-mechanical techniques produced comparable bond strength values, whereas the chemo-mechanical technique resulted in the lowest performance. This outcome aligns with previous reports showing that gutta-percha solvents—particularly eucalyptol and chloroform—can adversely affect dentin adhesion [20,21]. The mechanism is likely related to the physicochemical alterations that these solvents induce on the dentin substrate. Solvent exposure may modify the organic matrix, alter the moisture balance required for resin infiltration, and leave residues that interfere with hybrid-layer formation, particularly when total-etch adhesive systems are used. Additionally, softened endodontic sealers and gutta-percha can remain compacted into the dentinal walls after retreatment, hindering tubule penetration and reducing micromechanical interlocking [22,23].

Among the solvents assessed, eucalyptol caused the most pronounced reduction in bond strength across the cervical, middle, and apical thirds. Eucalyptol’s oily consistency and lower volatility mean it is more likely to leave hydrophobic residues on dentin surfaces. Published studies indicate that such residues reduce surface energy and impede adhesive diffusion, offering a plausible explanation for the consistently lower bond strength recorded in the eucalyptol group. This effect appears more substantial in the apical third, where bonding is already challenged by anatomical constraints [24].

The regional differences observed in bond strength followed the expected coronal-to-apical gradient. The coronal third demonstrated the highest values, supported by its wider bonding surface area [25], higher dentinal tubule density, and lower mineralization [26], all of which promote resin tag formation and hybrid-layer development. In contrast, the apical third is characterized by fewer tubules, greater mineral content, and a more unfavorable cavity configuration with a high C-factor [25,27], which together restrict resin flow and polymerization efficiency. Furthermore, this region retains more sealer and gutta-percha remnants due to limited accessibility during cleaning and shaping [28], compounding the reduction in bond strength. Studies assessing bond strength operate on the premise that higher bond strength increases the likelihood of bonded surfaces withstanding functional loads, ultimately contributing to the longevity of restorations [29,30]. Although the reliability of bond strength tests in predicting clinical performance has been questioned, they remain a common method for evaluating adhesive performance [31]. In addition to measuring bond strength, evaluating failure modes offers valuable insight into the efficiency of the bonding system and highlights its weakest point [30]. In the present study, the push-out test was performed because it is the most appropriate method for measuring the retention of posts [15].

In this study, four distinct modes of failure were defined and analyzed. Each sample underwent two assessments by the first examiner, spaced two weeks apart, followed by an additional assessment by a second examiner to evaluate reliability and the coefficient of variation. Two samples from each group were sputter-coated with a thin layer of gold, mounted on stubs, and examined under SEM at a magnification of ×500 to investigate failure modes and the surface morphological structure. The classification of failure modes is subjective, as it relies on visual interpretation, examiner experience, and assessment conditions. Variability in defining adhesive, cohesive, and mixed failures can lead to inconsistencies between observers. While tools like SEM improve accuracy, human judgment still plays a role. Using multiple examiners and standardized criteria can help reduce subjectivity, but it remains an inherent challenge in bonding studies.

Adhesive T-C was the most common failure mode due to the challenges at this interface, where the smear layer and residual debris can hinder proper bonding between the cement and dentin. To support what was recently discussed, these factors create a weaker adhesion compared to the cement–post interface, making it more susceptible to failure.

The present in vitro study had some limitations. First, the in vitro design does not fully replicate the complex biological and mechanical conditions present in the oral environment. Additionally, only a single type of fiber post and luting cement was evaluated, which limits the generalizability of the findings. Moreover, the assessment of failure modes involved a degree of subjectivity, which may influence the interpretation of results.

## 5. Conclusion

Within the limitations of this study, the push-out bond strength of fiber posts was significantly influenced both by the gutta-percha removal technique and by the root canal level. The chemico-mechanical approach, which relies on solvents, may alter dentin structure and negatively affect the quality of adhesive bonding. Bond strength was highest in the coronal third, followed by the middle and apical thirds. Adhesive failure at the dentin–cement interface was the most common failure mode, highlighting the ongoing difficulty of achieving durable adhesion to root dentin. Although the differences observed were statistically significant, their magnitude was also considered in terms of clinical relevance to assess their potential impact on post retention and long-term restorative performance.

## Figures and Tables

**Table 1:** Types of failure mode and their descriptions.

Mode of Failure	Description
Adhesive failure at the resin cement–post interface	Most of the cement remains adhered to the dentin, with little to no cement on the post.
Adhesive failure at the resin cement–dentin interface	Cement remnants are predominantly found on the post, or no detectable luting cement remains on the dentin.
Cohesive failure	Cement remnants are present on both the post and the dentin, indicating internal failure within the cement layer.
Mixed failure	A combination of adhesive failures at both the resin cement–post and resin cement–dentin interfaces within the same specimen.

**Table 2:** Descriptive Statistics of Bond Strength (MPa).

Method	Coronal Section (Mean ± SD)	Middle Section (Mean ± SD)	Apical Section (Mean ± SD)	Overall (Mean ± SD)
Mechanical	9.26 ± 0.34	8.34 ± 0.37	7.28 ± 0.29	8.29 ± 0.88
Thermal-Mechanical	8.97 ± 0.37	8.81 ± 0.34	7.33 ± 0.24	8.37 ± 0.81
Chemical-Mechanical	5.97 ± 0.31	6.15 ± 0.31	5.92 ± 0.24	6.01 ± 0.30
Total	8.06 ± 1.54	7.77 ± 1.21	6.84 ± 0.71	7.56 ± 1.31

**Table 3:** Post Hoc Tukey HSD test of push-out bond strength between methods of RCFM removal and root sections.

Category	Subgroup	Mean ± SD (MPa)
Method	Mechanical	8.29 ± 0.88 ^A^
	Thermal-Mechanical	8.37 ± 0.81 ^A^
	Chemical-Mechanical	6.01 ± 0.30 ^B^
Root Section	Coronal	8.06 ± 1.54 ^c^
	Middle	7.77 ± 1.21 ^d^
	Apical	6.84 ± 0.71 ^e^

Different uppercase superscript letters indicate a significant difference between RCFM removal methods (*p* < 0.05). Different lowercase superscript letters indicate a significant difference between root sections (*p* < 0.05).

**Table 4:** Distribution of Mode of Failure (overall, by method, and by root section).

Mode of Failure	Frequency (N)	Percentage (%)	Methods			Root Sections		
			Mechanical	Thermal-Mechanical	Chemical-Mechanical	Coronal	Middle	Apical
Adhesive T-C	60	44.4%	19	20	21	23	18	19
Adhesive C-P	20	14.8%	9	6	5	7	9	4
Cohesive	35	25.9%	12	13	10	6	12	17
Mixed	20	14.8%	5	6	9	9	6	5
Total	135	100%	45	45	45	45	45	45

## Data Availability

The data of the current study are available from the corresponding author upon request.
